# Prevalence of associated extracardiac anomalies in prenatally diagnosed congenital heart diseases

**DOI:** 10.1371/journal.pone.0248894

**Published:** 2021-03-18

**Authors:** Chi-Son Chang, Sir-yeon Hong, Seo-yeon Kim, Yoo-min Kim, Ji-Hee Sung, Suk-Joo Choi, Soo-young Oh, Cheong-Rae Roh, Jinyoung Song, June Huh, I-Seok Kang

**Affiliations:** 1 Department of Obstetrics and Gynecology, Samsung Medical Center, Sungkyunkwan University School of Medicine, Seoul, Korea; 2 Department of Obstetrics and Gynecology, Chung-Ang University College of Medicine, Seoul, Korea; 3 Department of Pediatrics, Samsung Medical Center, Sungkyunkwan University School of Medicine, Seoul, Korea; University of Sydney, AUSTRALIA

## Abstract

**Objective:**

To investigate the prevalence of extracardiac anomalies (ECA) in prenatally diagnosed congenital heart diseases (CHD), and to provide more information for counseling of women with prenatally diagnosed fetal CHD.

**Methods:**

This was a retrospective cohort study of 791 cases of fetal CHD diagnosed by prenatal ultrasound from January 2005 to April 2018. Associated ECAs included extracardiac structural malformation (ECM), chromosomal anomaly, and 22q11.2 microdeletion. CHD was classified into 10 groups according to a modified anatomic and clinical classification of congenital heart defects.

**Results:**

The overall prevalence of ECA in our CHD cohort was 28.6% (226/791): ECM, 25.3%; chromosomal anomaly, 11.7%; and 22q11.2 microdeletion, 5.5%. For those with ECM, ventricular septal defect (VSD) had the highest prevalence (34.5%), followed by anomalies of atrioventricular junctions and valves (28.8%) and heterotaxy (26.9%). For those with chromosomal anomaly, anomalies of atrioventricular junctions and valves had the highest prevalence (37.5%), followed by anomalies of atria and interatrial communications (25.0%) and VSD (22.9%). 22q11.2 microdeletion was detected only in those with anomalies of extrapericardial arterial trunks (14.3%) or ventricular outflow tracts (6.4%).

**Conclusion:**

ECM, chromosomal anomaly, and 22q11.2 microdeletion have different prevalence according to the type of CHD.

## Introduction

Congenital heart disease (CHD) is the most frequently detected prenatal anomaly, and it is one of the most common causes of infant death [1,2]. A few decades ago, most CHDs were diagnosed after birth. However, recent advance in obstetric ultrasound has led to increased prenatal diagnosis of CHD. Prenatal diagnosis and referral to a tertiary center for postnatal treatment are important to improve outcome of CHD. The outcome of CHD depends on the type of CHD, the severity of CHD, and associated extracardiac anomalies (ECA).

CHD is often associated with major extracardiac malformations (ECM), chromosomal anomalies, and genetic syndromes [3]. Previous studies have shown that ECMs are found in 20–60% in live born CHD population [1,2,4,5]. It is known that ECMs associated with CHDs have significant impact on the clinical course of CHD. Patients with ECMs show worse outcomes, because they may require additional interventions independently of their cardiac pathology [2,3,6]. Chromosomal anomalies are present in 5–15% of live born CHD patients [2,4,5]. CHDs with chromosomal anomalies tend to have worse outcomes such as fetal demise in utero (FDIU), termination of pregnancy, and neonatal death [6–8]. 22q11.2 microdeletion syndrome, also known as CATCH22 syndrome, is a genetic disorder present in some patients with CHD. CHDs are present in 75–80% of patients with 22q11.2 microdeletion syndrome [9,10] and 22q11.2 microdeletion is found in 5–15% of patients with CHD [11–13].

For these reasons, it is important to know the risk of coexisting ECMs, chromosomal anomalies, and 22q11.2 microdeletion during the prenatal period to predict prognosis of patients with CHD and to establish plans for postnatal treatment. Thus, the objective of this study was to determine the prevalence of extracardiac anomalies (ECAs) including structural anomalies, chromosomal anomalies, and 22q11.2 microdeletion in patients prenatally diagnosed with fetal CHD in a single tertiary center in Korea.

## Materials and methods

This was a retrospective cohort study of pregnant women with fetal CHD at a single tertiary center (Samsung Medical Center, Seoul, Korea) between January 2005 and April 2018. Women who were diagnosed with fetal structural CHD by prenatal ultrasound at 16 weeks of gestation or more were included in this study. Exclusion criteria were: 1) normal fetal heart on follow-up ultrasound exam or neonatal echocardiogram, and 2) fetal arrhythmia without structural CHD. This study was approved by the Institutional Review Board of Samsung Medical Center for Clinical Research (No. 2019-03-135). IRB approval of this study was obtained prior to data collection. Informed consent was not necessary because this study was retrospective study using medical records of participants and data were analyzed anonymously.

Pregnant women who were suspected for fetal CHD underwent targeted ultrasound conducted by trained obstetricians and/or fetal echocardiograms conducted by pediatric cardiologists. Prenatal diagnosis of CHD was made based on obstetric targeted ultrasound and/or fetal echocardiograms. If fetal echocardiogram was not done, prenatal diagnosis was made based on results of obstetric targeted ultrasound. In the postnatal period, neonatal echocardiogram was done by pediatric cardiologists to confirm the final diagnosis of CHD. For those whose neonatal echocardiogram was unavailable due to follow up loss, termination of pregnancy, or fetal demise in utero, the final diagnosis was made based on prenatal diagnosis.

When fetal CHD was diagnosed, women were counseled for prenatal diagnosis, postnatal treatment, and prognosis of CHD. They were also counseled for the benefit and risk of prenatal invasive diagnostic test for chromosomal or genetic syndromes including 22q11.2 microdeletion syndrome. Amniocentesis, an invasive diagnostic test, was performed if the woman chose it. When prenatal genetic study was not performed, cord blood sample obtained during birth or neonatal peripheral blood sample was used for chromosomal analysis and/or 22q11.2 microdeletion analysis.

CHD was classified into 10 groups according to a modified anatomic and clinical classification of congenital heart defects (ACC-CHD) used in the epidemiology of children or fetuses with congenital heart defects (EPICARD) study: [14] 1) heterotaxy, 2) anomalies of the venous return, 3) anomalies of the atria and interatrial communications, 4) anomalies of the atrioventricular junctions and valves, 5) complex anomalies of atrioventricular connections, 6) functionally univentricular hearts, 7) ventricular septal defect (VSD), 8) anomalies of the ventricular outflow tracts, 9) anomalies of the extrapericardial arterial trunks, and 10) other unclassified anomalies.

Extracardiac anomaly (ECA) was defined as having ECM, chromosomal anomaly, or 22q11.2 microdeletion. ECM was classified into 8 groups: 1) central nervous system (CNS), 2) face and neck, 3) pulmonary, 4) gastrointestinal, 5) genitourinary, 6) skeletal, 7) multiple anomalies, and 8) others including fetal hydrops.

Prevalence of ECA, ECM, chromosomal anomaly, and 22q11.2 microdeletion were analyzed according to the type of CHD and their subtypes in the total study population, including live birth, follow-up loss, termination of pregnancy, and fetal death. The prevalence of each ECM group by the type of CHD was also analyzed. Only live birth cases with final diagnosis confirmed by postnatal echocardiogram were also analyzed for these prevalence.

Obtained data were analyzed using the Statistical Package for Social Sciences version 25 (SPSS Statistics; IBM, Armonk, NY, USA). Continuous variables were compared using independent-sample parametric (Student t-test) or nonparametric (Mann-Whitney U test) tests depending on data normality. Categorical variables were compared using Chi-square test or Fisher’s exact test when one or more expected value was less than 5. Results were considered statistically significant when *p* value was less than 0.05.

## Results

**Fig 1:**
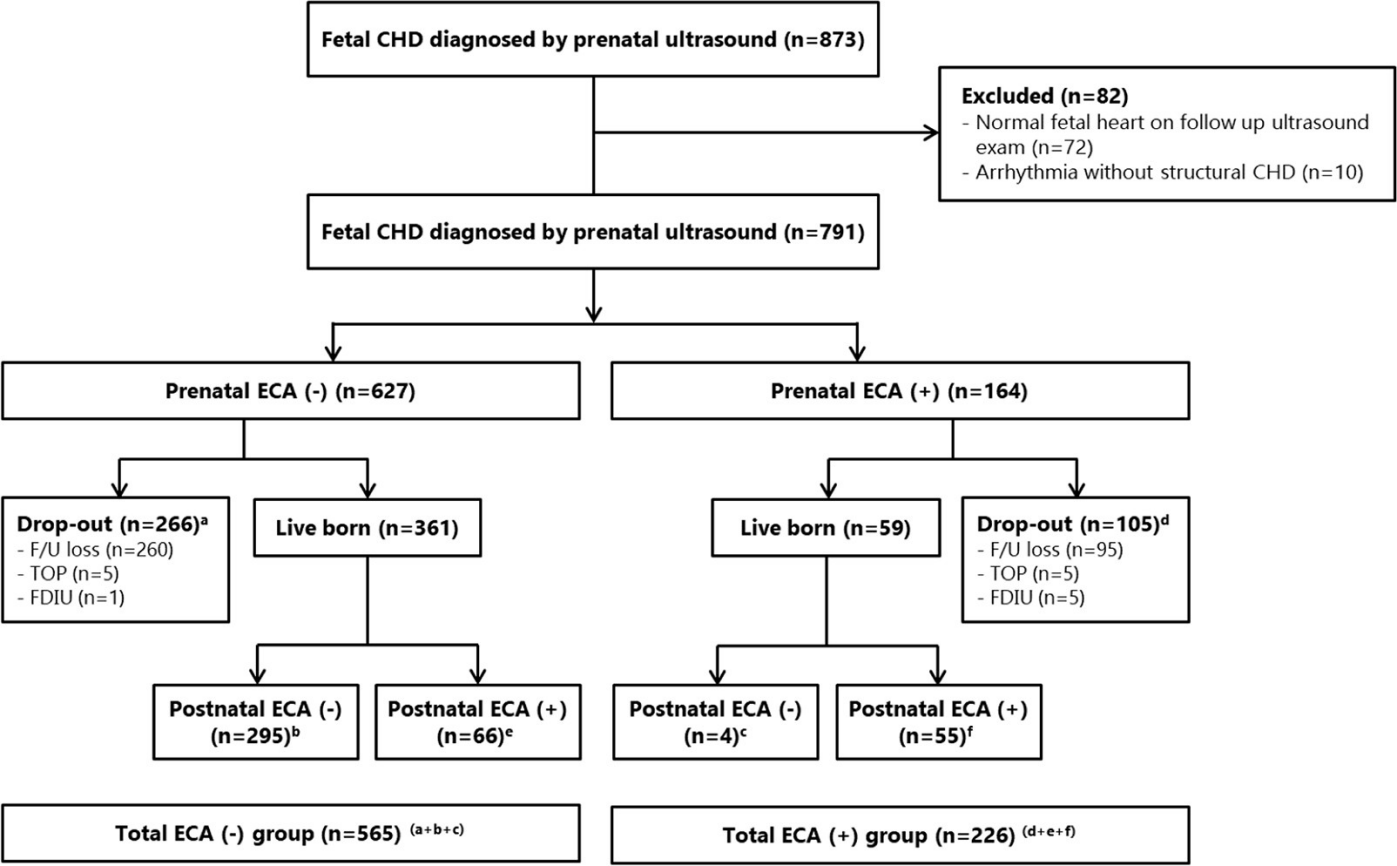
Study population and flowchart. CHD, congenital heart disease; TOP; termination of pregnancy; FDIU, fetal death in utero; ECA, extracardiac anomalies.

During the study period, 873 cases of fetal CHD were diagnosed by prenatal ultrasound. Among them, 72 cases showing normal fetal heart on follow-up ultrasound exam and 10 cases with fetal arrhythmia without structural CHD (6 cases with premature atrial contractions, 4 cases with atrioventricular block) were excluded (Fig 1). As a result, 791 cases were included in the analysis. Maternal characteristics are described in Table 1. Amniocentesis was done for 182 cases. Chromosomal anomalies were found in 21/182 (11.5%) and 22q11.2 microdeletion was found in 3/85 (3.5%). ECM was found in 147 (18.6%) cases by prenatal ultrasound. Of 791 cases included in the analysis, 627 (79.3%) had isolated CHDs and 164 (20.7%) cases had associated ECAs by prenatal ultrasound and amniocentesis. A total of 355 (44.9%) cases were lost to follow up. Ten (1.3%) cases chose termination of pregnancy and 6 (0.8%) cases resulted in FDIU. Cases of CHD with associated ECA had significantly higher maternal age and significantly lower gestational age at diagnosis of CHDs than cases of isolated CHD (Table 2). Rates of lost to follow-up, termination of pregnancy, and FDIU were significantly higher while live birth rate was significantly lower in cases with associated ECA than in cases with isolated CHD.

**Table 1:** Maternal characteristics.

Number of pregnant women	787
Maternal age (years)	31.8±3.8
Nullipara	454 (57.7)
Twin pregnancy	43 (5.5)
CHD in only one twin	39
CHD in both twins	4
Number of fetuses diagnosed with CHD	791
Gestational age at diagnosis of CHD (weeks)	24.7±4.9
Gestational age at delivery (weeks) (live birth only)	37.8±2.5

Data are shown in number (%) or mean ± standard deviation.

CHD, congenital heart disease.

**Table 2:** Pregnancy outcome of prenatally diagnosed congenital heart diseases according to extracardiac anomalies.

	Prenatal ECM^a^		p value	Prenatal chromosomal anomaly		p value	Prenatal 22q11.2 microdeletion		p value	Prenatal ECA^b^		p value
	(-)	(+)		(-)	(+)		(-)	(+)		(-)	(+)	
**Number**	644	147		161	21		82	3		627	164	
**Maternal age**	31.7±3.7	32.3±4.0	0.113	32.6±3.8	34.5±4.6	0.038	32.0±3.9	29.3±2.9	0.244	31.7±3.7	32.4±4.1	0.033
**GA at diagnosis**	24.9±4.9	23.3±4.5	<0.001	23.3±4.0	22.0±3.7	0.162	22.4±3.0	24.0±3.6	0.365	25.0±4.9	23.2±4.4	<0.001
**Lost to follow up**	271 (42.1)	84 (57.1)	0.001	63 (39.1)	12 (57.1)	0.115	27 (32.9)	2 (66.7)	0.267	260 (41.5)	95 (57.0)	<0.001
**Termination of pregnancy**	7 (1.1)	2 (2.0)	0.406	6 (3.7)	2 (9.5)	0.232	4 (4.9)	1 (33.3)	0.168	5 (0.8)	5 (3.0)	0.037
**Fetal death in utero**	1 (0.2)	5 (3.4)	0.001	5 (3.1)	0 (0)	1.000	1 (1.2)	0 (0)	>0.009	1 (0.2)	5 (3.0)	0.002
**Live birth**	365 (56.7)	55 (37.4)	<0.00	87 (54.0)	7 (33.3)	0.074	50 (61.0)	0 (0)	0.066	361 (57.6)	59 (36.0)	<0.001

Data are shown in number (%) or mean ± standard deviation.

ECM, extracardiac malformations; ECA, extracardiac anomalies; GA, gestational age.

^a^ extracardiac malformations defined as extracardiac structural malformation.

^b^ extracardiac anomalies defined as having any ECM or chromosomal anomaly or 22q11.2 microdeletion.

Among 420 (53.1%) live born infants, 121 (28.8%) had associated ECA and 299 (71.2%) had isolated CHD. Chromosomal anomalies, 22q11.2 microdeletion, and ECM were found in 22/219 (10.0%), 6/129 (4.7%), and 108 (25.7%) cases, including those who had genetic tests prenatally. Among 126 cases who did not test prenatal chromosomal study but tested in the postnatal period, 15 (11.9%) cases revealed to have chromosomal anomaly. Among 79 cases who did not test for 22q11.2 microdeletion prenatally but tested in the postnatal period, 22q11.2 microdeletion were found in 6 (7.6%) cases. The rates of chromosomal anomaly and 22q11.2 microdeletion were not significantly different between the prenatal and postnatal tests (chromosomal anomaly: 11.5% (prenatal) vs. 11.9% (postnatal), P = 0.922; 22q11.2 microdeletion: 3.5% (prenatal) vs. 7.6% (postnatal), P = 0.213). Among 164 cases who were found to have ECA during the prenatal period, 4 cases were diagnosed as isolated cases by postnatal examinations. Among 627 cases of prenatal isolated CHDs, 66 cases were found to have associated ECAs by postnatal examinations (Fig 1).

Prevalence of ECA, ECM, chromosomal anomaly and 22q11.2 microdeletion according to the type of CHD were analyzed in the total study population, including live birth, lost to follow up, termination of pregnancy, and FDIU (Table 3). In those with CHD, anomalies of ventricular outflow tracts were the most common (n = 303), followed by ventricular septal defect (VSD) (n = 110), anomalies of extrapericardial arterial trunks (n = 96), and functionally univentricular hearts (n = 84). The prevalence of ECA in the total CHD population was 28.6% (226/791). The prevalence of ECA was the highest in those with VSD (39.1%), followed by that of anomalies of atrioventricular junctions and valves (37.9%), unclassified anomaly (33.3%), and anomalies of atria and interatrial communications (29.6%). Prevalence of ECM, chromosomal anomaly, and 22q11.2 microdeletion were 25.3% (200/791), 11.7% (36/308), and 5.5% (9/164), respectively.

**Table 3:** Prevalence of extracardiac anomalies by type of congenital heart disease (total study population).

No	Type	N	ECM^a^	Chromosomal anomaly^b^	22q11.2 microdeletion^b^	ECA^c^
**1**	**Heterotaxy, including isomerism and mirror-imagery**	52	14 (26.9)	1/22 (4.5)	0/12 (0)	14 (26.9)
**2**	**Anomalies of the venous return**	28	6 (21.4)	0/4 (0)	0/0	6 (21.4)
**3**	**Anomalies of the atria and interatrial communications**	27	6 (22.2)	2/8 (25.0)	0/3 (0)	8 (29.6)
**4**	**Anomalies of the atrioventricular junctions and valves**	66	19 (28.8)	9/24 (37.5)	0/8 (0)	25 (37.9)
**5**	**Complex anomalies of atrioventricular connections**	10	0 (0)	0/4 (0)	0/0	0 (0)
**6**	**Functionally univentricular hearts**	84	13 (15.5)	3/28 (10.7)	0/15 (0)	14 (16.7)
**7**	**Ventricular septal defects**	110	38 (34.5)	8/35 (22.9)	0/11 (0)	43 (39.1)
**8**	**Anomalies of the ventricular outflow tracts**	303	75 (24.8)	11/145 (7.6)	6/94 (6.4)	86 (28.4)
**9**	**Anomalies of the extrapericardial arterial trunks**	96	24 (25.0)	2/35 (5.7)	3/21 (14.3)	25 (26.0)
**10**	**Other unclassified anomalies**	15	5 (33.3)	0/3 (0)	0/0	5 (33.3)
	**Total**	791	200 (25.3)	36/308 (11.7)	9/164 (5.5)	226 (28.6)

Data are shown in number (%).

ECM, extracardiac malformations; ECA, extracardiac anomalies.

^a^ extracardiac malformations, defined as extracardiac structural malformation.

^b^ denominators were those who underwent the examination during either prenatal or postnatal periods.

^c^ extracardiac anomalies, defined as having any ECM or chromosomal anomaly or 22q11.2 microdeletion.

Trisomy 21 (n = 13) and trisomy 18 (n = 7) were the two most common chromosomal anomalies. The prevalence of chromosomal anomaly was the highest in those with anomalies of atrioventricular junctions and valves (37.5%, 9/24), followed by that in those with anomalies of atria and interatrial communications (25.0%, 2/8) and VSD (22.9%, 8/35). 22q11.2 microdeletion was detected only in those with anomalies of extrapericardial arterial trunks (14.3%, 3/21) and ventricular outflow tracts (6.4%, 6/94). Detailed prevalence of ECA, ECM, chromosomal anomaly, and 22q11.2 microdeletion by the type of CHD and their subtypes are shown in S1 Table.

Table 4 shows the prevalence of each group of ECM by the type of CHD. Face and neck anomalies (16.0%) were the most common ECMs, followed by multiple (15.5%), genitourinary (15.0%), CNS (14.0%), skeletal (13.5%), and gastrointestinal (10.5%) anomalies. Those with VSD had the highest prevalence of ECM (34.5%), followed by those with unclassified anomaly (33.3%), anomalies of atrioventricular junctions and valves (28.8%), and heterotaxy (26.9%).

**Table 4:** Types of extracardiac malformation by type of congenital heart disease (total study population).

No	Type	N	ECM	CNS	Face and neck	Pulmonary	GI	GU	Skeletal	Multiple	Others
**1**	**Heterotaxy, including isomerism and mirror-imagery**	52	14 (26.9)	0 (0)	3 (21.4)	1 (7.1)	6 (42.9)	3 (21.4)	0 (0)	1 (7.1)	0 (0)
**2**	**Anomalies of the venous return**	28	6 (21.4)	0 (0)	0 (0)	1 (16.7)	1 (16.7)	1 (16.7)	3 (50.0)	0 (0)	0 (0)
**3**	**Anomalies of the atria and interatrial communications**	27	6 (22.2)	0 (0)	2 (33.3)	1 (16.7)	0 (0)	3 (50.0)	0 (0)	0 (0)	0 (0)
**4**	**Anomalies of the atrioventricular junctions and valves**	66	19 (28.8)	3 (15.8)	1 (5.3)	0 (0)	3 (15.8)	1 (5.3)	5 (26.3)	3 (15.8)	3 (15.8)
**5**	**Complex anomalies of atrioventricular connections**	10	0 (0)	0 (0)	0 (0)	0 (0)	0 (0)	0 (0)	0 (0)	0 (0)	0 (0)
**6**	**Functionally univentricular hearts**	84	13 (15.5)	3 (23.1)	3 (23.1)	0 (0)	2 (15.4)	2 (15.4)	0 (0)	1 (7.7)	2 (15.4)
**7**	**Ventricular septal defects**	110	38 (34.5)	8 (21.1)	4 (10.5)	5 (13.2)	2 (5.3)	5 (13.2)	3 (7.9)	7 (18.4)	4 (10.5)
**8**	**Anomalies of the ventricular outflow tracts**	303	75 (24.8)	9 (12.0)	16 (21.3)	4 (5.3)	6 (8.0)	11 (14.7)	11 (14.7)	14 (18.7)	4 (5.3)
**9**	**Anomalies of the extrapericardial arterial trunks**	96	24 (25.0)	3 (12.5)	3 (12.5)	2 (8.3)	1 (4.2)	3 (12.5)	5 (20.8)	5 (20.8)	2 (8.3)
**10**	**Other unclassified anomalies**	15	5 (33.3)	2 (40.0)	0 (0)	0 (0)	0 (0)	1 (20.0)	0 (0)	0 (0)	2 (40.0)
	**Total**	791	200 (25.3)	28 (14.0)	32 (16.0)	14 (7.0)	21 (10.5)	30 (15.0)	27 (13.5)	31 (15.5)	17 (8.5)

Data are shown in number (%).

ECM, extracardiac malformation; CNS, central nervous system; GI, gastrointestinal; GU, genitourinary.

Table 5 shows the prevalence of each type of CHD by ACC-CHD and their subgroups in only live birth cases whose final diagnosis was confirmed by postnatal echocardiogram. Ventricular outflow tracts anomalies (n = 169) were also the most common CHDs in these subjects, followed by anomalies of extrapericardial arterial trunks (n = 63), VSD (n = 47), and anomalies of atrioventricular junctions and valves (n = 37). The prevalence of ECA in CHD was 28.8% (121/420). Prevalence of ECM, chromosomal anomaly, and 22q11.2 microdeletion were 25.7% (108/420), 10.0% (22/219), and 4.7% (6/129), respectively. Those with anomalies of atria and interatrial communications had the highest prevalence of chromosomal anomaly (25.0%, 2/8), followed by those with anomalies of atrioventricular junctions and valves (23.5%, 4/17), VSD (14.3%, 3/21), and functionally univentricular hearts (11.8%, 2/17).

**Table 5:** Prevalence of extracardiac anomalies by type of congenital heart disease (live birth only).

No	Type	N	ECM^a^	Chromosomal anomaly ^b^	22q11.2 microdeletion ^b^	ECA ^c^
**1**	**Heterotaxy, including isomerism and mirror-imagery**	19	8 (42.1)	0/12 (0)	0/7 (0)	8 (42.1)
**2**	**Anomalies of the venous return**	14	3 (21.4)	0/3 (0)	0/0	3 (21.4)
**3**	**Anomalies of the atria and interatrial communications**	25	6 (24.0)	2/8 (25.0)	0/3 (0)	8 (32.0)
**4**	**Anomalies of the atrioventricular junctions and valves**	37	10 (27.0)	4/17 (23.5)	0/5 (0)	12 (32.4)
**5**	**Complex anomalies of atrioventricular connections**	5	0 (0)	0/2 (0)	0/0	0 (0)
**6**	**Functionally univentricular hearts**	31	5 (16.1)	2/17 (11.8)	0/12 (0)	5 (16.1)
**7**	**Ventricular septal defects**	47	12 (25.5)	3/21 (14.3)	0/7 (0)	14 (29.8)
**8**	**Anomalies of the ventricular outflow tracts**	169	43 (25.4)	10/107 (9.3)	3/76 (3.9)	50 (29.6)
**9**	**Anomalies of the extrapericardial arterial trunks**	63	17 (27.0)	1/30 (3.3)	3/19 (15.8)	17 (27.0)
**10**	**Other unclassified anomalies**	10	4 (40.0)	0/2 (0)	0/0	4 (40.0)
	**Total**	420	108 (25.7)	22/219 (10.0)	6/129 (4.7)	121 (28.8)

Data are shown in number (%).

ECM, extracardiac malformations; ECA, extracardiac anomalies.

^a^ extracardiac malformations, defined as extracardiac structural malformation.

^b^ denominators were those who underwent the examination during either prenatal or postnatal periods.

^c^ extracardiac anomalies, defined as having any ECM or chromosomal anomaly or 22q11.2 microdeletion.

## Discussion

This study revealed that the prevalence was 28.6% for overall ECA, 25.3% for ECM, 11.7% for chromosomal anomaly, and 5.5% for 22q11.2 microdeletion in those with prenatally diagnosed fetal CHD. These prevalence were much higher than those reported in previous studies [2,5,6]. Discrepancies in the prevalence of ECA between studies might be due to the lack of standardized definition of ECA, different study population in aspect of race and ethnicity, and different selection criteria. Bensemlali et al. [6] have reported that the prevalence was 18.6% for ECA, 11.7% for ECM, and 9.8% for genetic syndrome in 2,036 CHD fetuses. However, they excluded minor ECMs such as hypospadias and hexadactylia for analysis because of their limited clinical significance. Egbe et al. [2] have reported an even lower prevalence of ECA in a nationwide CHD population (n = 97,154) (prevalence: 13.6% for ECA, 11.4% for ECM, and 2.2% for genetic syndrome). The lower prevalence of ECA might be because these studies only included live birth CHD patients. In contrast, Tennstedt et al. [1] have analyzed 129 CHD fetuses sent for necropsy and reported that the prevalence is 66% for ECM and 33% for chromosomal anomalies. Based on the fact that almost all cases of prenatally diagnosed CHD visited a tertiary medical center, most women who were lost to follow-up might have terminated their pregnancies at other local clinics. According to former reports, existence of ECA has a strong influence on parental decision. It is related to a high rate of termination of pregnancy [7,8,15]. Knowing and having information about the risk of ECA in each type of CHD might be helpful in consultation of couples with prenatally diagnosed CHD fetus.

Our study included not only live birth CHD patients, but also follow-up loss, termination of pregnancy, and FDIU cases. When CHD was accompanied by ECA in prenatal examinations, the rate of lost to follow up and adverse pregnancy outcomes such as termination of pregnancy and FDIU was higher (64.0%, 105/164) than that in those with isolated CHDs (42.4%, 266/627). For this reason, inclusion of only live born infants might underestimate the prevalence of ECM and genetic abnormalities. However, the prevalence of ECA in the total population did not differ from that in only live birth population of CHD in our study. It might be due to several reasons. First, the detection rate of structural anomalies in antenatal ultrasound is low, ranging from 27% to 68% [16–18]. In our study, of 627 cases, there were 66 (10.5%) false negative cases. They showed no ECA in prenatal examination. However, they were confirmed to have ECA after birth. Second, due to the development of imaging and genetic test technique, detailed additional work-up for structural anomalies and genetic abnormalities can be done after birth. Further tests at postnatal period may reveal unknown pathology that could not be found in the prenatal period. Nevertheless, limitation of our study was that follow-up loss, termination of pregnancy, and FDIU cases were unable to undergo postnatal confirmative diagnosis of ECA. In addition, patients who were not diagnosed as CHD in fetal period but diagnosed after birth were not included in this study, because our study population was based on the prenatal ultrasound database in our hospital. Some CHDs such as small atrial septal defect or VSD might not have been diagnosed during prenatal period but diagnosed after birth. This might be one of the reason for the discrepancies in the prevalence of ECA between the previous studies and our study.

In our study, the prevalence of ECA was the highest in VSD, followed by that in anomalies of atrioventricular junctions and valves, unclassified anomaly, and anomalies of atria and interatrial communications. The prevalence of ECM was also the highest in VSD, followed by that in unclassified anomaly, anomalies of atrioventricular junctions and valves, and heterotaxy. These findings were consistent with those of previous studies [1,3,6].

In our study, face and neck anomalies were the most common ECMs, followed by multiple, genitourinary, CNS, skeletal, and gastrointestinal anomalies. According to Egbe et al. [2], craniofacial, respiratory, and genitourinary malformations were associated with CHD, although they did not report incidence of ECM by type of CHD. CHD had different prevalence by ECM type. For example, gastrointestinal anomaly was the most common ECM in heterotaxy while CNS anomaly was the most frequent one in VSD. However, the number of each type of ECM was so small that it was difficult to obtain clinical meaning.

In the study of Tennstedt et al. [1], chromosomal anomalies were found in AVSD (62%, 13/21), tetralogy of Fallot (TOF) (50%, 2/4), coarctation of aorta (CoA) (42.9%, 3/7), and VSD (42%, 15/36) in the order of frequency. Egbe et al. [2] found that septal defect was most frequently found in these with genetic syndrome. Although classification of CHD is different between studies, we also found that septal defects such as AVSD and VSD showed high prevalence of chromosomal anomaly in common.

The prevalence of 22q11.2 microdeletion was 5.5% in our study. Because of the strong correlation of 22q11.2 microdeletion with cardiac anomaly, some obstetricians recommend routine screening for 22q11.2 microdeletions when CHD is diagnosed on prenatal ultrasound. However, while certain types of CHD have frequent prevalence of 22q11.2 microdeletions, some cardiac defects are rarely accompanied by 22q11.2 microdeletion [13,19]. In our study, 22q11.2 microdeletion was detected only in two categories of CHD, anomalies of extrapericardial arterial trunks and ventricular outflow tracts (4/33 in TOF and variants, 1/2 in truncus arteriosus, 1/3 in TOF type double outlet right ventricle (DORV), 1/4 in CoA, 1/2 in interruption of artic arch (IAA), and 1/1 in right aortic arch). In the study of Lee et al. [13], which was done in a single tertiary center in Korea similar to our study, 22q11.2 microdeletion was found in 4.7% (53/1,137) of fetuses diagnosed with CHD prenatally during the study period. The spectrum of the type of CHD was 24 (45%) in TOF, 10 (19%) in IAA, 5 (9%) in VSD, 4 (8%) in DORV, and 4 (8%) in CoA in their study, consistent with our study. Conotruncal cardiac anomalies are the most common cardiovascular anomalies in those with 22q11.2 microdeletion syndrome. Conotruncal cardiac anomalies include TOF, truncus arteriosus, and IAA. Right aortic arch, aberrant subclavian artery, and major aortopulmonary collateral arteries are also known to be frequently associated with CHD accompanied by 22q11.2 microdeletion. On the other hand, cardiac anomalies such as corrected transposition of great arteries (TGA), Ebstein’s anomaly, aortic valve stenosis, and hypoplastic left heart syndrome (HLHS) are rarely associated with 22q11.2 microdeletion [19]. When selecting candidate for genetic test of 22q11.2 microdeletion in women with prenatally diagnosed cardiac anomaly, conotruncal anomaly associated with aortic arch or ductus arteriosus anomalies should increase the suspicion of a positive test result.

The major strength of our study was that we analyzed a large number of CHD patients over a decade, including not only live birth patients, but also prenatally diagnosed cases with follow-up loss, termination of pregnancy, or fetal demise. However, this can be considered a limitation as well, depending on the perspective, because the diagnosis of CHD and ECA may change during the postnatal period. Therefore, we have analyzed only live birth cases whose final diagnosis was confirmed by postnatal examinations and the prevalence of ECA, ECM, chromosomal anomaly, and 22q11.2 microdeletion in only live birth cases were not significantly different from those in the total population. This study also has some limitations. First, this study was mainly based on medical charts. Its retrospective nature might cause information bias. Second, since our institution is a tertiary center, potential selection bias and generalizability problem may be present. Third, patients who were not diagnosed as CHD in fetal period but diagnosed after birth were not included in this study. Fourth, diagnostic techniques has evolved and persons performing the study have changed over the long time period, and therefore, this may potentially affected the detection rate of prenatal CHD. Last, only a small proportion of total CHD population were tested for chromosomal anomalies and 22q11.2 microdeletion in both prenatal and postnatal periods. Among total CHD population, only 38.9% (308/791) underwent chromosomal examination, and only 20.7% (164/791) underwent test for 22q11.2 microdeletion during either prenatal or postnatal periods. Although the lower rates of tests might have affected the results, the rates of chromosomal anomaly and 22q11.2 microdeletion were not significantly different between the prenatal and postnatal tests.

## Conclusion

ECM, chromosomal anomaly, and 22q11.2 microdeletion had different prevalence according to the type of CHD. Results of this study might offer valuable information when counseling women with prenatally diagnosed CHD.

## Supporting information

S1 Table Prevalence of extracardiac anomalies by type of congenital heart disease (total study population). (DOCX)
