# Supplementary material for: Prevalence of associated extracardiac anomalies in prenatally diagnosed congenital heart diseases
Source: PLoS One. 2021 Mar 18;16(3):e0248894. doi: 10.1371/journal.pone.0248894 (PMC7971844; doi:10.1371/journal.pone.0248894)
Supplement: S1 Table — (DOCX) [file pone.0248894.s001.docx]

**S1 Table. Prevalence of extracardiac anomalies by type of congenital heart disease (total study population).**

| **No** | **Type** | **N** | **ECM**^a^ | **Chromosomal anomaly**^b^ | **22q11.2 microdeletion**^b^ | **ECA**^c^ |
| --- | --- | --- | --- | --- | --- | --- |
| **1** | **Heterotaxy, including isomerism and mirror-imagery** | **52** | **14 (26.9)** | **1/22 (4.5)** | **0/12 (0)** | **14 (26.9)** |
|  | 1.1 Situs inversus totalis | 5 | 1 (20.0) | 0/3 | 0/1 | 1 (20.0) |
|  | 1.2 Right isomerism | 30 | 5 (16.7) | 0/11 | 0/6 | 5 (16.7) |
|  | 1.3 Left isomerism | 6 | 3 (50.0) | 0/3 | 0/2 | 3 (50.0) |
|  | 1.4 Situs ambiguous, visceral heterotaxy | 11 | 5 (45.5) | 1/5 | 0/3 | 5 (45.5) |
| **2** | **Anomalies of the venous return** | **28** | **6 (21.4)** | **0/4 (0)** | **0/0** | **6 (21.4)** |
|  | 2.1 Persistent left superior vena cava | 19 | 3 (15.8) | 0/2 | 0/0 | 3 (15.8) |
|  | 2.2 Total anomalous pulmonary venous return | 5 | 1 (20.0) | 0/2 | 0/0 | 1 (20.0) |
|  | 2.3 Partial anomalous pulmonary venous return | 4 | 2 (50.0) | 0/0 | 0/0 | 2 (50.0) |
| **3** | **Anomalies of the atria and interatrial communications** | **27** | **6 (22.2)** | **2/8 (25.0)** | **0/3 (0)** | **8 (29.6)** |
|  | 3.1 Cor triatrium | 2 | 0 (0) | 0/0 | 0/1 | 0 (0) |
|  | 3.2 Atrial septal defect | 24 | 6 (25.0) | 2/8 | 0/2 | 8 (33.3) |
|  | 3.3 Atrial septal aneurysm | 1 | 0 (0) | 0/0 | 0/0 | 0 (0) |
| **4** | **Anomalies of the atrioventricular junctions and valves** | **66** | **19 (28.8)** | **9/24 (37.5)** | **0/8 (0)** | **25 (37.9)** |
|  | 4.1 Congenital anomalies of the tricuspid valve | 24 | 7 (29.2) | 1/4 | 0/1 | 8 (33.3) |
|  | Ebstein’s anomaly | 13 | 4 (30.8) | 0/0 | 0/0 | 4 (30.8) |
|  | Dysplastic tricuspid valve | 4 | 1 (25.0) | 0/1 | 0/0 | 1 (25.0) |
|  | Tricuspid regurgitation (isolated) | 7 | 2 (28.6) | 1/3 | 0/1 | 3 (42.9) |
|  | 4.2 Congenital anomalies of the mitral valve (mitral regurgitation) | 1 | 0 (0) | 0/1 | 0/0 | 0 (0) |
|  | 4.3 Atrioventricular septal defects (AVSD) | 41 | 12 (29.3) | 8/19 | 0/7 | 17 (41.5) |
|  | Complete AVSD | 23 | 7 (30.4) | 5/11 | 0/3 | 10 (43.5) |
|  | Complex AVSD^d^ | 13 | 5 (38.5) | 2/5 | 0/3 | 6 (46.2) |
|  | Partial AVSD | 5 | 0 (0) | 1/3 | 0/1 | 1 (20.0) |
| **5** | **Complex anomalies of atrioventricular connections** | **10** | **0 (0)** | **0/4 (0)** | **0/0** | **0 (0)** |
|  | 5.1 Congenitally corrected transposition of the great arteries | 10 | 0 (0) | 0/4 | 0/0 | 0 (.0) |
| **6** | **Functionally univentricular hearts** | **84** | **13 (15.5)** | **3/28 (10.7)** | **0/15 (0)** | **14 (16.7)** |
|  | 6.1 Double-inlet ventricle | 3 | 0 (0) | 0/2 | 0/1 | 0 (0) |
|  | Double-inlet left ventricle | 1 | 0 (0) | 0/1 | 0/0 | 0 (0) |
|  | Double-inlet right ventricle | 2 | 0 (0) | 0/1 | 0/1 | 0 (0) |
|  | 6.2 Absence of one atrioventricular connection | 15 | 2 (13.3) | 2/6 | 0/3 | 3 (20.0) |
|  | Mitral atresia | 2 | 1 (50.0) | 1/2 | 0/1 | 1 (50.0) |
|  | Tricuspid atresia | 13 | 1 (7.7) | 1/4 | 0/2 | 2 (15.4) |
|  | 6.3 Left ventricular hypoplasia (hypoplastic left heart syndrome) | 39 | 6 (15.4) | 0/9 | 0/4 | 6 (15.4) |
|  | 6.4 Right ventricular hypoplasia | 27 | 5 (18.5) | 1/11 | 0/7 | 5 (18.5) |
|  | Pulmonary atresia with intact ventricular septum (IVS) | 22 | 3 (13.6) | 1/9 | 0/6 | 3 (13.6) |
|  | Hypoplastic right heart syndrome | 5 | 2 (40.0) | 0/2 | 0/1 | 2 (40.0) |
| **7** | **Ventricular septal defects (VSD)** | **110** | **38 (34.5)** | **8/35 (22.9)** | **0/11 (0)** | **43 (39.1)** |
| **8** | **Anomalies of the ventricular outflow tracts** | **303** | **75 (24.8)** | **11/145 (7.6)** | **6/94 (6.4)** | **86 (28.4)** |
|  | 8.1 Transposition of the great arteries (TGA) | 51 | 13 (25.5) | 1/25 | 0/18 | 13 (25.5) |
|  | TGA with IVS | 18 | 4 (22.2) | 0/12 | 0/12 | 4 (22.2) |
|  | TGA with VSD | 18 | 4 (22.2) | 0/7 | 0/2 | 4 (22.2) |
|  | Complex TGA^e^ | 15 | 5 (35.7) | 1/6 | 0/4 | 5 (35.7) |
|  | 8.2 Other abnormal ventriculo-arterial connections | 58 | 13 (22.4) | 1/27 | 1/17 | 14 (24.1) |
|  | Double outlet right ventricle (VSD type) | 19 | 7 (36.8) | 1/10 | 0/5 | 7 (36.8) |
|  | Double outlet right ventricle (TOF type) | 21 | 4 (19.0) | 0/10 | 1/7 | 5 (23.8) |
|  | Double outlet right ventricle (TGA type) | 18 | 2 (11.1) | 0/7 | 0/5 | 2 (11.1) |
|  | Double outlet left ventricle | 0 | 0 (0) | 0/0 | 0/0 | 0 (0) |
|  | 8.3 Tetralogy of Fallot (TOF) and variants | 142 | 32 (22.5) | 7/76 | 4/52 | 41 (28.9) |
|  | TOF | 113 | 22 (19.5) | 4/57 | 3/39 | 29 (25.7) |
|  | Pulmonary atresia with VSD | 28 | 9 (32.1) | 3/18 | 1/13 | 11 (39.3) |
|  | Absent pulmonary valve syndrome | 1 | 1 (100) | 0/0 | 0/0 | 1 (100) |
|  | 8.4 Anomalies of the intrapericardial arterial trunks | 8 | 3 (37.5) | 0/2 | 1/2 | 4 (50.0) |
|  | Common arterial trunk (truncus arteriosus) | 7 | 2 (28.6) | 0/2 | 1/2 | 3 (42.9) |
|  | Aorto-pulmonary window | 1 | 1 (100) | 0/0 | 0/0 | 1 (100) |
|  | 8.5 Left ventricular outflow tract and aortic valvar anomalies | 16 | 4 (25.0) | 0/4 | 0/2 | 4 (25.0) |
|  | Aortic stenosis | 14 | 3 (21.4) | 0/2 | 0/1 | 3 (21.4) |
|  | Bicuspid aortic valve | 1 | 0 (0) | 0/1 | 0/0 | 0 (0) |
|  | Aortic atresia | 1 | 1 (100) | 0/1 | 0/1 | 1 (100) |
|  | 8.6 Right ventricular outflow tract and pulmonary valvar anomalies | 28 | 10 (35.7) | 2/11 | 0/3 | 10 (35.7) |
|  | Pulmonary stenosis | 27 | 10 (37.0) | 2/10 | 0/3 | 10 (37.0) |
|  | Dysplastic pulmonary valve | 1 | 0 (0) | 0/1 | 0/0 | 0 (0) |
| **9** | **Anomalies of the extrapericardial arterial trunks** | **96** | **24 (25.0)** | **2/35 (5.7)** | **3/21 (14.3)** | **25 (26.0)** |
|  | 9.1 Coarctation of the aorta | 51 | 14 (27.5) | 2/23 | 1/11 | 15 (29.4) |
|  | Coarctation of the aorta | 48 | 13 (27.1) | 2/21 | 0/9 | 14 (29.2) |
|  | Aortic arch hypoplasia (tubular) | 3 | 1 (33.3) | 0/2 | 1/2 | 1 (33.3) |
|  | 9.2 Interruption of the aortic arch | 16 | 5 (31.3) | 0/8 | 1/6 | 5 (31.3) |
|  | 9.3 Anomalies of the aortic arches | 26 | 4 (15.4) | 0/4 | 1/4 | 4 (15.4) |
|  | Double aortic arch | 8 | 0 (0) | 0/1 | 0/1 | 0 (0) |
|  | Right aortic arch | 18 | 4 (22.2) | 0/3 | 1/3 | 4 (22.2) |
|  | 9.4 Anomalies of the pulmonary artery | 3 | 1 (33.3) | 0/0 | 0/0 | 1 (33.3) |
|  | Pulmonary arterial hypoplasia | 3 | 1 (33.3) | 0/0 | 0/0 | 1 (33.3) |
| **10** | **Other unclassified anomalies** | **15** | **5 (33.3)** | **0/3 (0)** | **0/0** | **5 (33.3)** |
|  | 10.1 Cardiac tumor | 9 | 4 (44.4) | 0/2 | 0/0 | 4 (44.4) |
|  | 10.2 Ductal aneurysm | 5 | 1 (20.0) | 0/0 | 0/0 | 1 (20.0) |
|  | 10.3 Aortic aneurysm | 1 | 0 (0) | 0/1 | 0/0 | 0 (0) |
|  | **Total** | **791** | **200 (25.3)** | **36/308 (11.7)** | **9/164 (5.5)** | **226 (28.6)** |

Data are shown in number (%).

ECM, extracardiac malformations; ECA, extracardiac anomalies.

^a^ extracardiac malformations, defined as extracardiac structural malformation.

^b^ denominators were those who underwent the examination during either prenatal or postnatal periods.

^c^ extracardiac anomalies, defined as having any ECM or chromosomal anomaly or 22q11.2 microdeletion.

^d^ AVSD with other anomalies including TOF, PS, PA, AS, CoA, TAPVR, DORV, PLSVC.

^e^ TGA with other anomalies including CoA, TAPVR, PS, ASD.
